# Nanostructured 3D Constructs Based on Chitosan and Chondroitin Sulphate Multilayers for Cartilage Tissue Engineering

**DOI:** 10.1371/journal.pone.0055451

**Published:** 2013-02-20

**Authors:** Joana M. Silva, Nicole Georgi, Rui Costa, Praveen Sher, Rui L. Reis, Clemens A. Van Blitterswijk, Marcel Karperien, João F. Mano

**Affiliations:** 1 3B's Research Group – Biomaterials, Biodegradables and Biomimetics, University of Minho, Headquarters of the European Institute of Excellence on Tissue Engineering and Regenerative Medicine, Taipas, Guimarães,Portugal; 2 ICVS/3B's – PT Government Associate Laboratory, Braga/Guimarães, Portugal; 3 Department of Tissue Regeneration, MIRA – Institute for Biomedical Technology and Technical Medicine, University of Twente, Enschede, The Netherlands; 4 Department of Developmental BioEngineering, MIRA – Institute for Biomedical Technology and Technical Medicine, University of Twente, Enschede, The Netherlands; Instituto de Engenharia Biomédica, University of Porto, Portugal

## Abstract

Nanostructured three-dimensional constructs combining layer-by-layer technology (LbL) and template leaching were processed and evaluated as possible support structures for cartilage tissue engineering. Multilayered constructs were formed by depositing the polyelectrolytes chitosan (CHT) and chondroitin sulphate (CS) on either bidimensional glass surfaces or 3D packet of paraffin spheres. 2D CHT/CS multi-layered constructs proved to support the attachment and proliferation of bovine chondrocytes (BCH). The technology was transposed to 3D level and CHT/CS multi-layered hierarchical scaffolds were retrieved after paraffin leaching. The obtained nanostructured 3D constructs had a high porosity and water uptake capacity of about 300%. Dynamical mechanical analysis (DMA) showed the viscoelastic nature of the scaffolds. Cellular tests were performed with the culture of BCH and multipotent bone marrow derived stromal cells (hMSCs) up to 21 days in chondrogenic differentiation media. Together with scanning electronic microscopy analysis, viability tests and DNA quantification, our results clearly showed that cells attached, proliferated and were metabolically active over the entire scaffold. Cartilaginous extracellular matrix (ECM) formation was further assessed and results showed that GAG secretion occurred indicating the maintenance of the chondrogenic phenotype and the chondrogenic differentiation of hMSCs.

## Introduction

Articular cartilage is an avascular, alymphatic, aneural, anisotropic tissue with limited capacity to regenerate [1], [2]. Due to these articular cartilage properties, tissue engineering approaches are needed to treat millions of people which suffer from traumatic injuries and degenerative cartilage diseases. A wide range of clinical options emerged to repair these lesions such as micro-fracture, micro-drilling auto and allografts, among others [3]. However, these treatments present some limitations, e.g. availability of sufficient cells for repair, quality and quantity of repaired tissue, and thereby fail to produce long-lasting repair [4], [5].

Tissue Engineering (TE) has appeared as a new method, which offers advantages when compared with current treatments [5], [6]. Scaffolds play an important role in TE strategies because they provide the initial support structure, guiding the differentiation and development of the cartilaginous tissue [7]–[9]. Typically native tissues exhibit a hierarchical organization from the nano- to the macro-scale levels which is difficult to achieve in conventional scaffolds. Thus, the control from the nano-sizes to macroscale of scaffold is of great interest because offers the possibility of developing structures with further capabilities. These capabilities include the fabrication of hierarchical-organized structures, the control of cell behaviour at the nano-level and the inclusion of other functionalities, such as the possibility of incorporate bioactive molecules, or tune the mechanical and degradation behaviour of the scaffold. This structures can be achieved by layer-by-layer (LbL) methodology, a versatile technique that permits to fabricate nanostructured multilayered films using a variety of polyelectrolytes [10]–[12]. The principle of this technique is based on alternate deposition of polyelectrolytes that will self-organize on the material surface [10]–[13]. The main application of LbL is the build-up of polyelectrolytes multilayers (PEMs) onto flat surfaces [10]–[12]. Just a few works reported the use of LbL to fabricate scaffolds. Such technique may be used to coat free-packet leachable spherical templates [14] or to agglomerate beads [15], leading in both cases to porous structures. In this work we propose the use of an LbL based bottom-up approach to produce three-dimensional (3D) highly porous scaffolds with a nanostructured organization reminiscent of the native extracellular matrix components of cartilage.

Cartilage specific ECM components play an important role in chondrogenesis as well as supporting the chondrogenic phenotype. Among the wide range of materials that has been explored for cartilage TE approaches appears chitosan (CHT) and chondroitin sulphate [6], [16]. CHT, a naturally derived is an excellent candidate for polycation due to its structural characteristics similar to glycosaminoglycan's (GAGs) and ability to support chondrogenic activity as well as Cartilage ECM expression by chondrocytes [17], [18]Chondroitin sulphate (CS) has a high negative charge density and it is the major GAG component of native cartilage tissue and it is reported its benefits for osteoarthritis as well as its ability to increase the production of ECM matrix a and capacity to induce the differentiation of multipotent stromal cells [1], [19]–[21]. This combination was already used in LbL methodology, however from our knowledge we reported from the first time the use of these polyelectrolytes for a 3D porous construct only based in PEMs for cartilage TE approaches [22].

The aim of this work is to prepare nanostructured 3D constructs, based on the LbL methodology, studying its effect on cartilage TE. For the proof of concept the build-up of CHT/CS PEMs onto flat surfaces was firstly characterized using quartz crystal microbalance (QCM). The biological performance was evaluated with a cell culture of primary bovine chondrocytes (BCH). The biological performance of highly porous nanostructured 3D scaffolds was also evaluated using BCH and multipotent bone marrow derived stromal cells (hMSCs). The maintenance of chondrogenic phenotype and the differentiation of hMSCs were also investigated.

## Materials and Methods

### Materials

Chitosan (CHT) of medium molecular weight (M_w_ 190–310 kDa, 75–85% degree of deacetylation, viscosity 200–800 cP) and chondroitin-4-sulphate (CS) (M_w_ 50–100 kDa) were purchased from Sigma Aldrich. Chitosan was purified by recrystallization. Paraffin wax spheres with ∅ 200 μm were purchased from Jojoba Desert Whale (Tucson, USA) and then modified with polyethylene imine (PEI) (Sigma- Aldrich, Mw 750 000). Glass coverslips with ∅13 mm (L4097–3) were purchased from Agar Scientific. Lysozyme from chicken egg white (lyophilized powder ≈10000 U/mg stored at 4°C) and hyaluronidase Type VIII (300 U/mg stored at −20°C) were purchased from Sigma- Aldrich.

### Methods

#### CHT/CS film build-up

The build-up process of CHT/CS PEMs was followed in situ by quartz crystal microbalance with dissipation monitoring (QCM-Dissipation, Q-Sense, Sweden), using a gold coated sensor excited at a fundamental frequency of 5 MHz and at seventh overtone (35 MHz). The crystals were cleaned in an ultrasound bath at 30°C using successive acetone, ethanol and isopropanol. Adsorption took place with a constant flow rate of 50 μL min^−1^.

The CHT (0.15% (w/v) in 1% acetic acid/ 0.15 M NaCl, pH = 5.5) solution was pumped into the system for 10 min to allow the adsorption equilibrium at the crystal surface. After rinsing with 0.15 M NaCl (10 min), the same procedure was followed for the deposition of CS (0.15% (w/v) in 1% acetic acid/ 0.15 M NaCl, pH = 5.5). The steps were repeated to the desire number of layers. The frequency and dissipation were monitored in real time. The thickness of the film was estimated using the Voigt model through the Q-Tools Software, from Q-Sense [23].

#### LbL assembly in 2D surfaces

The CHT/CS PEMs were deposited onto glass coverslips. The glass coverslips were placed in 70% (v/v) ethanol for 2 hours and then immersed in 0.15 M NaCl for 10 min. After these two steps the glass coverslips were dried using nitrogen flow. The multilayered film build-up started by immersing first the substrate in CHT during 10 min followed by the immersion in 0.15 M NaCl solution during 5 min. Then the coverslips were dipped in CS solution for 10 min, followed by immersion in 0.15 M NaCl over 5 min. These four steps allowed the assembling of one double layer. The process was repeated until 10 double layers were achieved.

#### Scaffolds production by LbL

The PEMs were constructed onto free-packet paraffin spheres previously modified with PEI. Paraffin spheres modified with PEI were chosen as the porogen and 150 mg of them placed into a modified cylindrical container, with a porous base. Drop wise addition of polyelectrolyte solutions and washing solutions over the top of assembly was done to form 10 double layers. The coated structure was placed in dichloromethane (DCM) to leach out the paraffin. After the leaching the samples were freeze dried.

#### Morphology

The morphology of the scaffolds after the leaching process and immersion in DCM was assessed by optical microscopy, using the Axioplan Imager Z1 microscope (Zeiss). Freeze-dried scaffolds were also observed by scanning electronic microscopy (SEM), using a Philips XL 30 ESEM-FEG operated at 15 kV accelerating voltage. Surface morphology of the coated glass coverslips was also observed using the same equipment at 7.5 kV accelerating voltage. All the samples were sputtered with a conductive gold layer, using a sputter coater (Cressington) for 40 s at a current of 40 mA.

#### Fourier transform infrared (FTIR) spectroscopy

FTIR measurements were recorded using an IRPrestige-21 spectrophotometer, by averaging 34 individual scans over the range 4400 cm^−1^ to 400 cm^−1^. The samples were prepared in potassium bromide (KBr) discs.

#### Swelling test

The water uptake ability of the scaffolds with known weight was determined by soaking them in phosphate buffered saline solution (PBS, Gibco) at pH = 7.4 up to 3 days at 37°C. The swollen scaffolds were removed at predetermined time points (t = 15 min, 30 min, 1 h, 2 h, 3 h, 4 h, 5 h, 1 day, 2 days and 3 days). After removing the excess water using a filter paper (Whatman Pergamyn Paper), the scaffolds were weighed with an analytical balance (Scaltec, Germany). The water uptake was calculated, where W_w_ and W_d_ are the weights of swollen and dried scaffold, respectively.
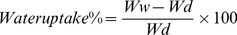
(1)


#### Enzymatic Degradation

The enzymatic degradation test was performed to evaluate the degradation profile of the scaffolds in simulated physiological environments. Scaffolds were placed at 37°C in PBS solution (pH = 7.4) or in enzymatic solution containing 2 mg.ml^−1^ of lysozyme and 0.33 mg.ml^−1^ of hyaluronidase (pH 7.4) (19). PBS and enzymatic solution were changed every third day (19). At predetermined time intervals, t = 3, 7 and 14 days the scaffolds were washed with distilled water to remove the salts. Then the scaffolds were immersed in ethanol 100% and dried for 1 day at room temperature. The percentage of weight loss (W_L_) was calculated, where W_i_ and W_f_ are the weights of dry scaffold and after incubation in PBS or enzymatic solution, respectively.
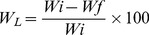
(2)


#### Mechanical Test

Compression tests were carried out using dynamic mechanical analysis (DMA), using Tritec 2000B equipment (Triton Technology, UK) to characterize the mechanical properties of cylindrical scaffolds in both the dry and wet states. The sizes of the samples were measured using a digital micrometre. Prior to any measurements in the wet state the scaffolds were immersed in PBS until equilibrium was reached. The measurement was carried out at 37°C under full immersion of the sample in liquid bath (PBS) placed in a Teflon® reservoir. Experiments were carried out in compression mode following cycles of increasing frequency ranging from 0.1 to 15 Hz, with constant strain amplitude of 30 μm. The frequency range chosen covers the characteristic timescales of the periodic loads felt by the scaffold *in vivo* (e.g. typical frequency of skeletal movement). The high frequency limit used in this study should provide information about the viscoelastic properties for the equivalent of short times [24].

#### Bovine articular chondrocytes and human mesenchymal stem cells culture

BCH cells were harvested from a patellar-femoral groove of calf legs and isolated by 0.2% collagenase overnight digestion (37°C) [25]. hMSCs were selected by adherence from the bone marrow of human donors undergoing total hip replacement [26]. Ethical approval has been obtained from a local medical ethical committee. The isolated BCH were washed, centrifuged and re-suspended in chondrocyte proliferation medium containing dulbecco's modified eagle medium (DMEM, Invitrogen, USA), fetal bovine serum (FBS, 10%, Sigma-Aldrich), non-essential aminoacids (0.1 mM, Sigma-Aldrich), penicillin/streptomycin (100 U/100 μg.mL^−1^, Invitrogen), proline (0.4 mM, Sigma-Aldrich) and Ascorbic acid 2-phosphate (ASAC, 0.2 mM, Invitrogen) in a humidified atmosphere with 5% CO_2_ and at 37°C. hMSCs were also washed, centrifuged and re-suspended in MSCs proliferation medium containing alpha modified eagle's medium (α-MEM, Invitrogen, USA), fetal bovine serum (FBS, 10% Sigma-Aldrich), penicillin/streptomycin (100 U/100 μg.mL^−1^, Invitrogen), Glutamine (2 mM, Sigma-Aldrich), basic fibroblast growth factor (bFGF, 1 ng.mL−1, Sigma Aldrich) and ASAC (0.2 mM, Invitrogen) in a humidified atmosphere with 5% CO2 and at 37°C. BCH and hMSCs were seeded in tissue culture flasks and the medium was change every third day until cells achieved 80% of confluence. BCH were used at passage 2 and hMSCs at passage 3. Prior to cell seeding scaffolds were sterilized with 70% (v/v) ethanol overnight and then rinsed three times in PBS, whereas surfaces were treated with ultraviolet (UV) light for 10 min to avoid the damage of the coating. Scaffolds and flat surfaces were then immersed for 4 hours in the medium appropriate for each cell type. For the scaffolds the seeding was performed by applying the cell suspension, with a concentration of 0.5×10^6^ cells in 25 μL of medium (per scaffold). For surfaces the cell concentration was adjusted to 1.32×10^4^ cells in 25 μL of medium (per glass coverslips). After cell attachment for 2 hours (37°C in a 5% CO_2_), chondrocytes proliferation medium, MSCs proliferation medium or differentiation medium (DMEM, 2 mM glutamine (Gibco), 0.2 mM ASAC (Invitrogen), 100 μg.mL^−1^ penicillin/Streptomycin (Invitrogen), 0.4 mM proline (Sigma-Aldrich), 100 μg.mL^−1^ sodium pyruvate (Sigma-Aldrich) and 50 mg/mL insulin-Transferrin-selenite (ITS+premix, BD biosciences), 10 ng.mL^−1^ TGFβ-3 (R&D systems) and 0.1 μM dexamethasone (Sigma-Aldrich)) was added.

#### Cell viability

Cell viability and morphology were assessed with live/dead assay, MTT assay and SEM analysis. The scaffolds were cut in half in order to perform live/dead and MTT assays at 1, 3, 14 and 21 days. Scaffolds were further observed by SEM. For the surfaces the live dead assay was performed at 1, 3, 7, 14 and 21 days followed by SEM visualization. Medium was changed every third day to maintain an adequate supply of cell nutrients.

#### Live/dead assay

To perform this assay the proliferation medium was aspirated from the wells in which the scaffolds and surfaces were deposited. The scaffolds and surfaces were then incubated with ethidium homodimer-1 (4 μM) and calcein-AM (2 μM) in PBS for 30 min at 37°C in a 5% CO_2_ atmosphere incubator. After 30 min the samples were immediately examined under an inverted fluorescent microscope (Nikon Eclipse E600) using Fluorescein isothiocyanate (FITC) and Texas Red Filter, as well as the NIS element-F.30 software.

#### MTT assay

The scaffolds were incubated in 900 μL of proliferation medium and 100 μL of MTT solution (5 mg.mL^−1^) per well for 2 h at 37°C in 5% CO_2_. MTT staining images were captured using a stereomicroscope with colour camera (Nikon SMZ-10A) and the Qcapture software.

#### Scanning electron microscopy observation

The structures with cells were fixed in formalin (10%) and dehydrated using serial concentrations of ethanol [70%, 80%, 90%, 96% and 100% (v/v), 30 min in each], before performing critical point drying (Balzers CPD 030). The samples were then coated with a conductive layer. The SEM observations were performed in a Philips XL 30 ESEM-FEG operated at 7.5–15 kV accelerating voltage.

#### DNA quantification

Scaffolds seeded with BCH and hMSCs in differentiation medium at 1, 14 and 35 days were washed with PBS and frozen at −80°C before proteinase K (Sigma Aldrich) digestion. Then the scaffolds were digested with 1 mg/mL of proteinase K in tris (hydroxymethyl) aminomethane ethylenediaminetetraacetic (Tris\EDTA) buffer (pH = 7.6) containing 18.5 μg.mL^−1^ idoacetamide and 1 μg/mL pepstatin A (Sigma Aldrich) at 56°C for 20 hours. Quantification of total DNA in each sample was determined with CyQuant DNA kit according to manufacturer description (Molecular probes, Eugene, Orgeon, USA), using a spectrofluorometer (Victor3, Perkin-Elmer, USA) at an emission wavelength of 520 nm and an excitation wavelength of 480 nm.

#### Histology

Haematoxylin & eosin (H&E) and alcian blue staining was used to analyse cell distribution and cartilage tissue formation, respectively. For histology analysis, scaffolds were fixed overnight in 10% formalin, and then dehydrated using sequential ethanol series [70%, 80%, 90%, 96%, and 100% (v/v), 30 min in each]. Once dehydrated, they were incubated in butanol overnight at 4°C and then in a solution of paraffin at 56°C for 12 hours. Sections of 4.5 μm were cut using a microtome (MicroM HM355S). After deparaffinization with xylene and rehydration using a graded ethanol series [from 100% to 70% (v/v)], the samples were stained using an automatic stainer (MicroM HMS740). For H&E staining samples were stained with haemotoxylin for 1 min and rinsed up to 6 min before being stained with eosin for 30 s. For alcian blue staining the samples were placed in alcian blue solution (0.5%, pH = 1) for 30 min and rinsed with tap water or distilled water for 4 min. In the last step nuclear fast red was added for 5 min before dehydration. Slides were assembled with resinous medium and mounted slides were examined under a light of Axioplan Imager Z1 microscope (Zeiss). Representative images were captured using a digital camera (AxioCAM MRCE) and treated using Axiovision software. Each assay was performed after 1, 14, 21 and 35 days of BCH and hMSCs culture, as well as with CHIT/CS scaffold as control.

#### Statistical Analysis

The experiments were carried out in triplicate unlike otherwise specified. The results were presented as mean ± standard deviation (SD). Statistical analysis was performed using one way ANOVA followed by Turkey test (Graph Pad Prism 5.0 for Windows). Statistical significance was set to p<0.05 (*) and p<0.01 (**).

## Results and Discussion

### CHT/CS film build-up

The build-up mechanism of the polymeric multilayered films made of CS and CHT was assessed in situ with QCM-D. This technique detected the adsorbed mass of polyelectrolytes and measured the viscoelastic properties of the surface [27], [28].

**Figure 1:**
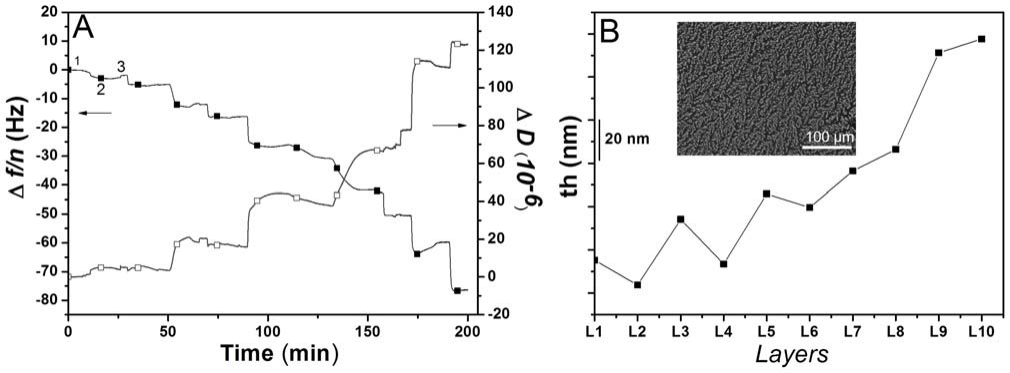
Build-up monitoring of the CHT/CS polyelectrolyte multi-layered using QCM for film constructed. A) Normalized frequency (?f_7_/7) and dissipation changes (?D_7_) obtain at 35 MHz, 1) deposition of CHT, 2) washing step and 3) deposition of CS; B) Estimated thickness (th) evolution and SEM micrographs of the multilayer surface with 10 double layers (inset image).

Figure 1A shows the build-up of 10 layers of CHT and CS in terms of variations on normalized frequency, ?f_n_/n (where n is the frequency overtone) and dissipation, ?D_7_. As expected, the normalized frequency decreased with each CHT and CS solutions injection, reflecting the increased mass over the gold sensor. The increase of ?D_7_ was due to the non-rigid adsorbed layer structure of the deposited film. During the washing step after the injection of each polyelectrolyte, the change of both ?f_7_/7 and ?D_7_ were relatively small compared to the total frequency variation associated to the adsorption of the respective polymer. This indicated a strong association of the layers on the surface of the crystal.

The combination of ?f_7_/7 and ?D_7_ gave information about the adsorbed amount and the variations of the viscoelastic properties [28]–[30]. The thickness of the film was estimated using the Voigt Model [23]. Figure 1B showed the thickness variation along the deposition of 10 layers. The results revealed a decrease of thickness from the first layer to the second one, which could be explained due to changes in water absorption [31]. The absorption of water was due to the presence of some groups in the polysaccharides (hydroxyl, carboxyl and sulphate groups) that interacted favourably with water molecules [31]. When the second layer was adsorbed the presence of opposite charge led to electrostatic interactions between them and the counterion-polymer. Consequently, water-polymer bonds were disrupted, resulting in an effective decrease of the hydrated film thickness [30]–[32]. The trend was observed during the first three pairs of layers. After the first three dL, this trend was no longer observed: there was an increase of thickness with the addition of CS. The SEM microphotography of the multilayered surface revealed a homogenous coating along the 2D flat surface (inset image of Figure 1B). Moreover, the surface presented a rough texture and some granularity, with characteristic diameter sizes around 2 μm as measured by Image J.

The results obtained through QCM measurements and SEM demonstrated that CS could be successfully used with CHT to conceive a homogeneous viscoelastic polymeric self-assembled coating using the LbL approach.

### Multilayer surface

Using LbL methodology it was possible to produce surfaces with tuned properties [10]–[12].In this work, multilayers of CHT and CS were prepared on glass coverslips by using the LbL methodology, obtaining self-assembled films with 10 double layers.

#### Cell behaviour in multilayers

**Figure 2:**
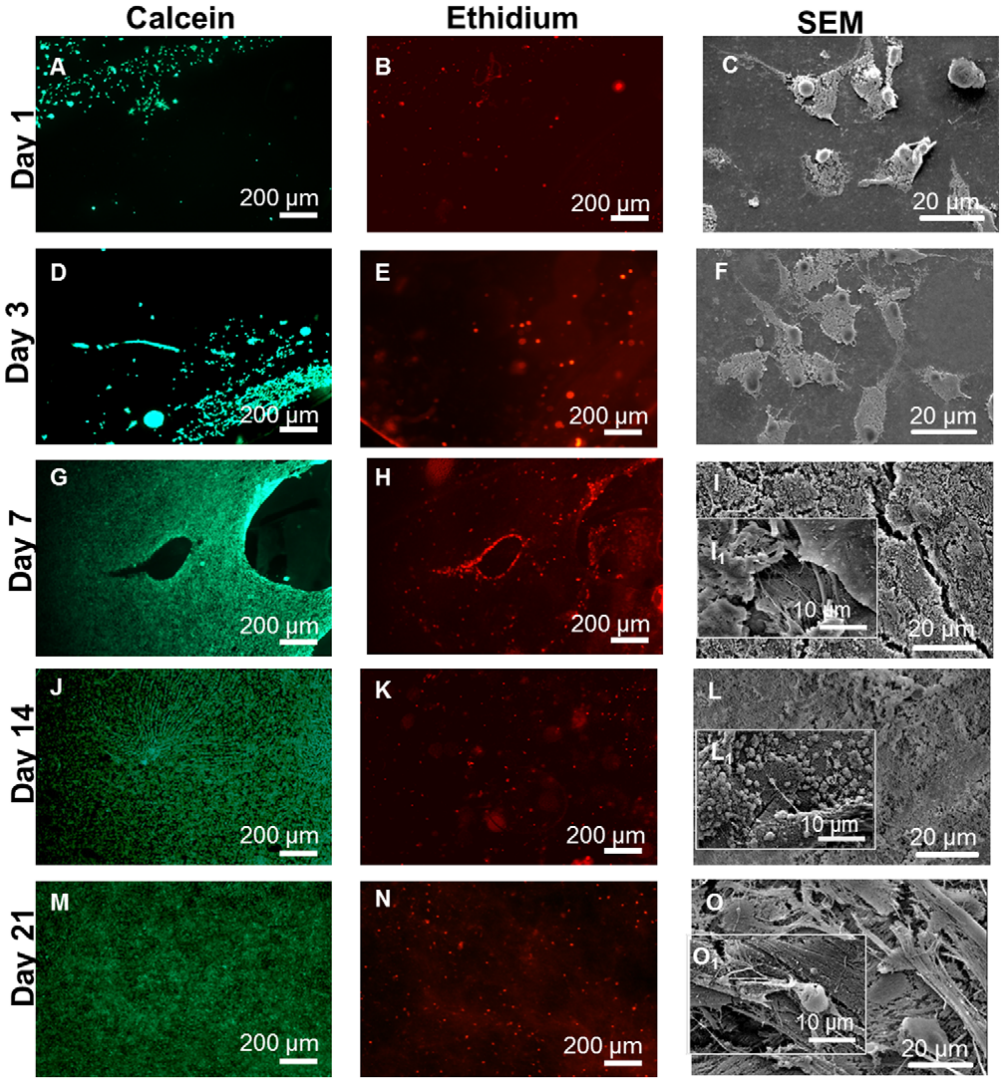
Live/dead assay and SEM micrographs of BCH seeded on glass coverslips coated with chitosan and chondroitin sulphate at day 1 (A, B, C), 3 (D, E, F), 7 (G, H, I), 14 (J, K, L) and 21 (M, N) of culture in proliferation medium.

In order to assess the cell viability in the surfaces, live/dead assay was performed (Figure 2A, 2B, 2D, 2E, 2G, 2H, 2J, 2K, 2M and 2N). The results showed a large amount of living cells and an increase in terms of cell number which results in cell confluence and in a continuous staining of calcein. The cell adhesion/morphology was also studied using SEM (Figure 2C, 2F, 2I, 2L and 2O). The results revealed that the BCH were attached to the surface from the earliest time points onwards. Attachment, adhesion and spreading are the first phase of cell/material interaction and the quality of this stage influenced the capacity of cells to proliferate and differentiate itself on contact with an implant [33], [34]. With increasing culture time, cells started to spread out along the surface, losing their round phenotype which might occur due to the high proliferation of cells or to the 2D environment that leads to de-differentiation. As a result of significant cell proliferation most of the surface area was already covered with cells after 7 days of culture. At 14 days cellular confluence was achieved.

These results suggested the potential of CHT and CS as polyelectrolytes for the fabrication of the 3D nanostructure.

### Nanostructured Scaffolds: physicochemical characterization

#### Scaffold preparation and morphology

**Figure 3:**
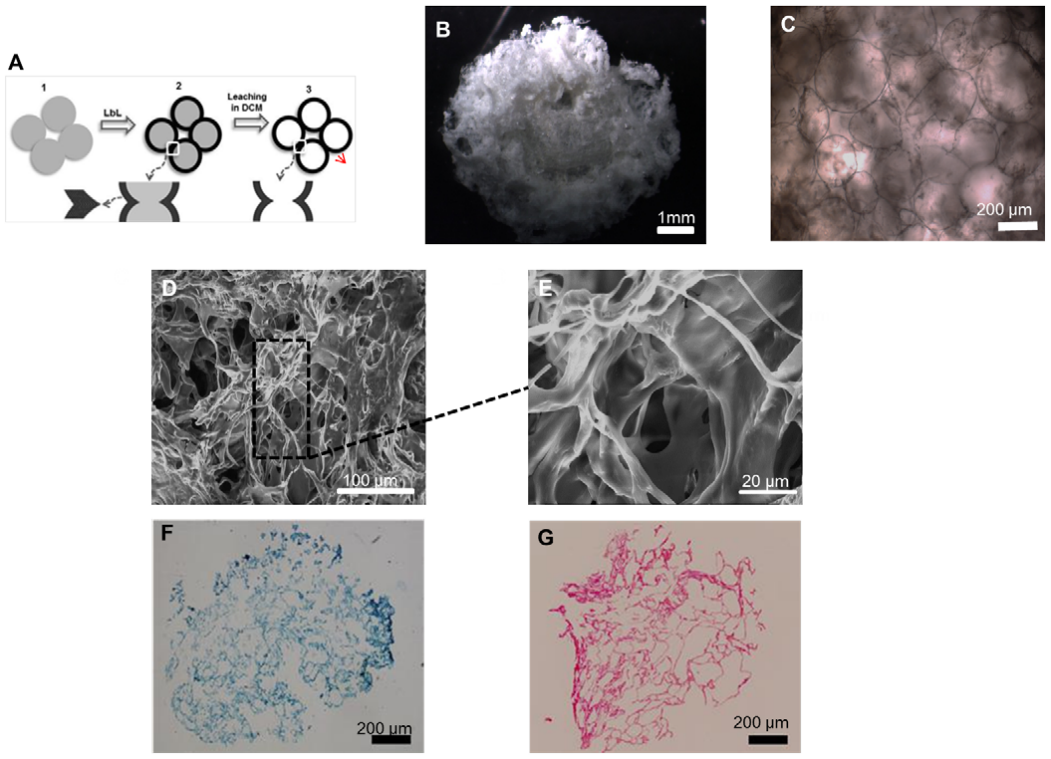
Scaffold characterization. A) Production steps of scaffolds: LbL and leaching of free-packet paraffin spheres, B)Digital photograph of the scaffold after all the steps C) Optical Microscopy image of the scaffolds after the leaching of the core material, D, E) SEM micrographs of cross-sections (two different magnifications) and Histological cross-sections of the scaffolds after staining with alcian blue (F) and eosin (G).

The use of bottom-up approaches to produce 3D porous structures is of particular interest to TE due to the hierarchical organization of the native tissues. It has been hypothesized that an interconnected 3D porous structure could be prepared combining LbL with leaching of free-packet paraffin spheres. A drop-wise addition method of PEMs over the 3D template formed by free-packet paraffin spheres was applied. This technique allows the formation of a 3D lattice arrangement from a randomly placed paraffin spheres. After coating the paraffin template was leached out and void spaces were created. Thus, the remaining material should be entirely composed by the CHT/CS multilayers (Figure 3A and Figure 3B).

The morphology of the obtained scaffolds after the leaching was observed by optical microscopy. The results clearly revealed a bubble-like morphology with geometry and pore sizes consistent with the paraffin spheres used as the template (Figure 3C). The paraffin spheres used as porogen had a diameter of 200 μm which is appropriate for cartilage TE approaches, allowing the deposition of ECM and cell infiltration even after the commonly shrinking after the freeze drying process [22]. The interconnectivity should be assured by the existence of physical contact points between the neighbouring paraffin beads that will result in a passage point between the two pores after the leaching process (see red arrow in Figure 3A). Further structural information was obtained by SEM (Figure 3D and 3E). SEM images of freeze-dried scaffolds revealed a noticeable hollow imprint of the porous spherical wax template morphology. This concept allowed the production of highly porous structure with controlled pore size and interconnectivity. Consequently, this type of scaffolds should allow the diffusion of substances as well as the integration of cells, namely its infiltration, migration and distribution in the entire volume of the scaffold. The histological cross-sections of the freeze-dried scaffold stained by alcian blue (Figure 3F) and eosin (Figure 3G) showed a homogeneous distribution of the polysaccharides. Alcian blue stained chondroitin sulphate [35] and eosin chitosan due to the high ability of this polysaccharide to adsorb anionic dyes [36].

#### Fourier transform infrared spectroscopy

**Figure 4:**
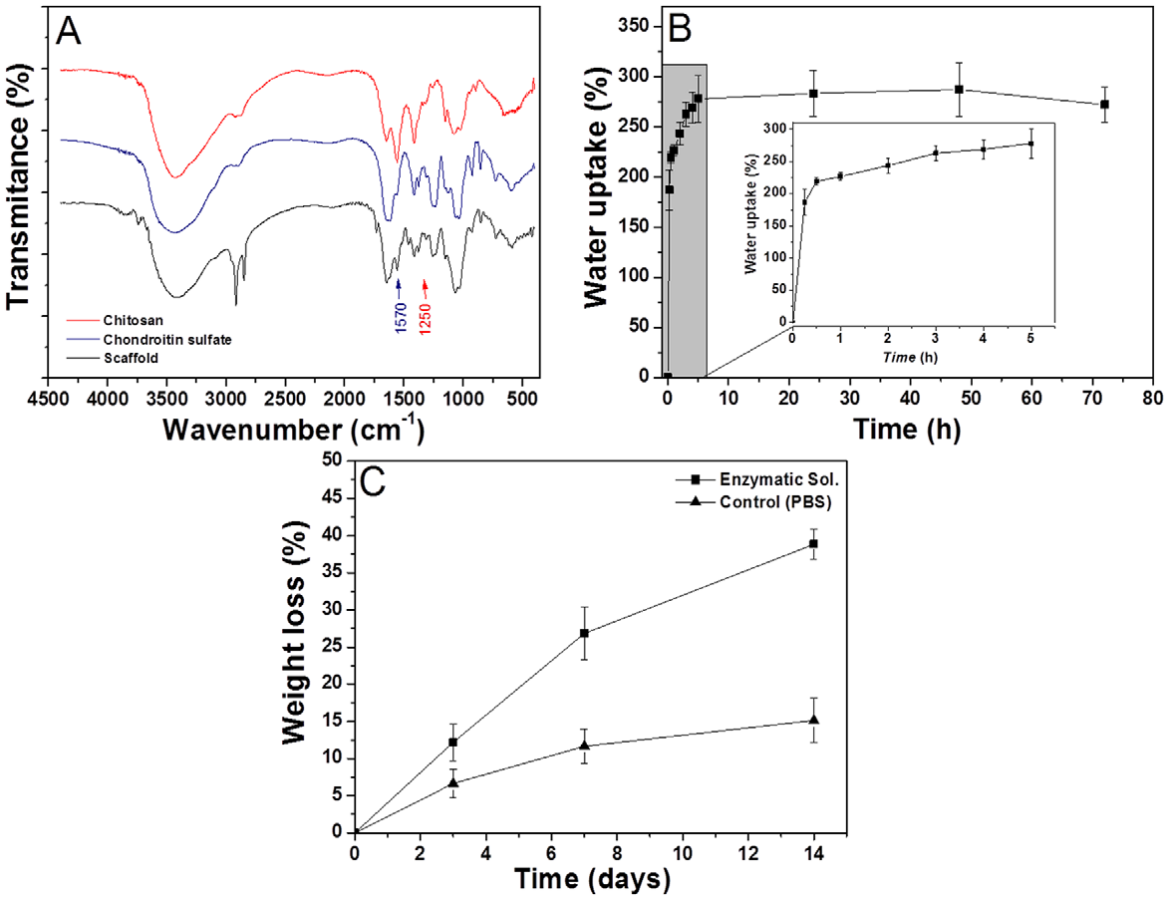
Physicochemical characterization of scaffolds. A) FTIR measurements of CHT/CS scaffolds and pure polysaccharides (CHT and CS), B) Swelling test up to 3 days (The inset graphic expands the water uptake for the first 5 hours), C) Weight loss of the scaffolds in PBS (▴) and in an enzymatic solution at 37°C (▪).

FTIR measurements (Figure 4A) were performed on the scaffold produced, as well as on both CHT and CS powders in order to identify the presence of both polysaccharides in the entire specimen. The spectra of CHT and CS were very similar, as expected, reflecting the similarities in the chemical structure of both materials. As a result, they shared some common peaks around 3400 cm-1 corresponding to –OH and N-H bond stretching vibrations, and the peaks around 2900 cm-1 corresponding to C-H stretching. Between 1020 cm-1 and 1080 cm-1 the peaks associated with the stretching of C-O bonds could be observed also. Moreover, the amide groups appeared at 1648 cm-1 [37].

In the CHT spectrum the amine group bonds, characteristic of this polysaccharide, appeared at 1570 cm-1 [18]. The representative peak of chondroitin sulphate was detected at 1250 cm-1 corresponding to the stretching in the S = O bond (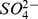) [37]. The spectrum of the scaffold showed globally the absorption peaks from both CHT and CS which was indicative of the presence of both raw materials in the final structure.

#### Swelling ability

Water uptake is particularly important for implantable materials because it allows the diffusion and exchange of nutrients and waste through the entire scaffold; moreover the water uptake ability also influences the mechanical performance of the biomaterial [9]. The materials used in the scaffold have abundant hydrophilic groups, such as hydroxyl, amino, sulphate and carboxyl groups, which can promote the swollen state of the scaffold [38]–[40]. The swelling ability was evaluated by soaking scaffolds in PBS (pH 7.4) at 37°C for 3 days (Figure 4B). The results showed that the water uptake increased mainly in the first hour and then tended to remain stable, reaching an equilibrium after 5 h (water uptake = 280%). This result could be explained with the high density of charge that increased the difference in osmotic pressure between the scaffold network and medium, resulting in a swollen scaffold. Moreover, the swelling ability of cartilage is well known to be highly dependent on the binding of water to polar groups of GAGs, namely carboxylate and sulphate groups, on electrostatic repulsion of GAGs and entropic contributions resulting from the mixing of water and counterions [41].

#### Enzymatic degradation

The biodegradability profile of scaffolds will dictate the changes in the structure that will occur upon the implantation. Enzymatic activity plays a fundamental role in the degradation of polysaccharides in vivo [42]. In vitro enzymatic degradation tests were performed with lysozyme and hyaluronidase solution and compared with weight loss in PBS (control). These two enzymes were chosen because they are present in the synovial fluid and they have as well the ability to cleave the polysaccharides used in this study [43], [44]. Lysozyme is able to degrade CHT and hyaluronidase has the ability to degrade both CHT and CS [44]–[47]. The weight loss as a function of time is presented in Figure 4C.

The results showed that the scaffolds degraded in the presence of the selected enzymes, showing weight losses of ca. 40% after 14 days. The degradation of scaffolds in the presence of the enzymatic solution was likely facilitated by the high porosity and interconnectivity of the structures allowing the easy access of the enzyme to their substrate. Moreover, the high hydrophilicity of scaffold (revealed by the high water uptake) could increase the interaction of scaffolds with the enzymatic solution, promoting the weight loss. The scaffolds placed in PBS also suffered some weight loss of ca. 15% after 14 days. In this case the weight loss could be the result of some disaggregation of the multilayered structure, as the polyelectrolytes were self-assembled through electrostatic interactions. The ions present in PBS may destabilize the structure and promote partial detachment between the macromolecules resulting in their release to the medium.

#### Mechanical Properties

**Figure 5:**
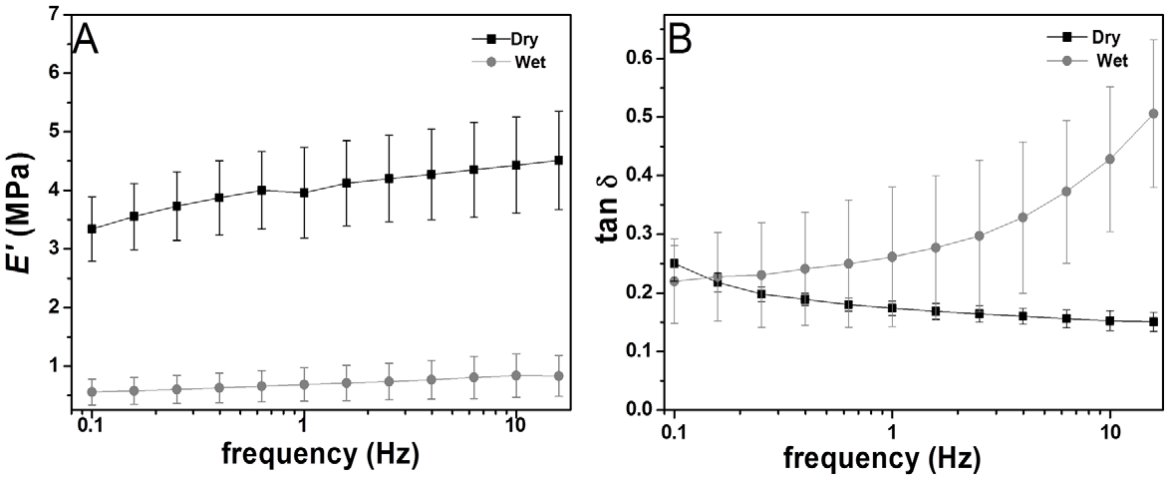
Variations of (A) Storage modulus (E') and (b) loss factor (tanδ) of the CHT/CS scaffolds obtained by LbL methodology. Experiments are reported for dry samples (▪) and hydrated samples in PBS at 37°C (•).

The viscoelastic/mechanical properties of an implantable device are fundamental for its performance *in vivo* [24]. Dynamical mechanical analysis (DMA) is an adequate non-destructive tool to characterize the mechanical and viscoelastic properties of polymeric materials [48], [49]. Since articular cartilage often is exposed to dynamic compression forces, DMA experiments were performed in a hydrated environment and at 37°C allowing the assessment of the mechanical properties of the scaffolds in more realistic conditions [24]. The storage modulus (E') and loss factor (tan δ) as a function of frequency of the developed scaffolds, in the dry and wet state are presented in Figure 5. The results for the hydrated scaffold showed a slight increase in both E' and tan δ with increasing frequency. In the dry state the values of E' were about one order of magnitude higher when compared with the wet state. This was consistent with the high water uptake ability of the scaffolds and the plasticization effect of water molecules in such kind of polysaccharides increasing their molecular mobility and decreasing the stiffness of the material. A similar loss of the stiffness due to the effect of water was observed in CHT membranes [50]. In both cases no evident variation of E' along the frequency axis were seen, indicating that no relaxation phenomena took place in the scaffolds within the time scale covered by the experiments. The tan δ of the dry sample decreased slightly with an increasing of frequency. However an opposite trend was observed when the samples were immersed in PBS. tan δ was higher in the wet samples, indicating that some dragging of entrapped water participated in energy loss for the hydrated structure [51]. The DMA results demonstrated viscoelastic behaviour of the scaffold which approached the viscoelastic nature of native cartilage [52]. Moreover, the E' values obtained in the wet state (0.6–0.8 MPa, 0.01 – 10 Hz) were included in the range of mandibular condylar cartilage E' values (0.1–1.5 MPa, 0.01 – 10 Hz ) [53].

### Cell behaviour in nanostructured scaffolds

#### Cell viability, adhesion and morphology

**Figure 6:**
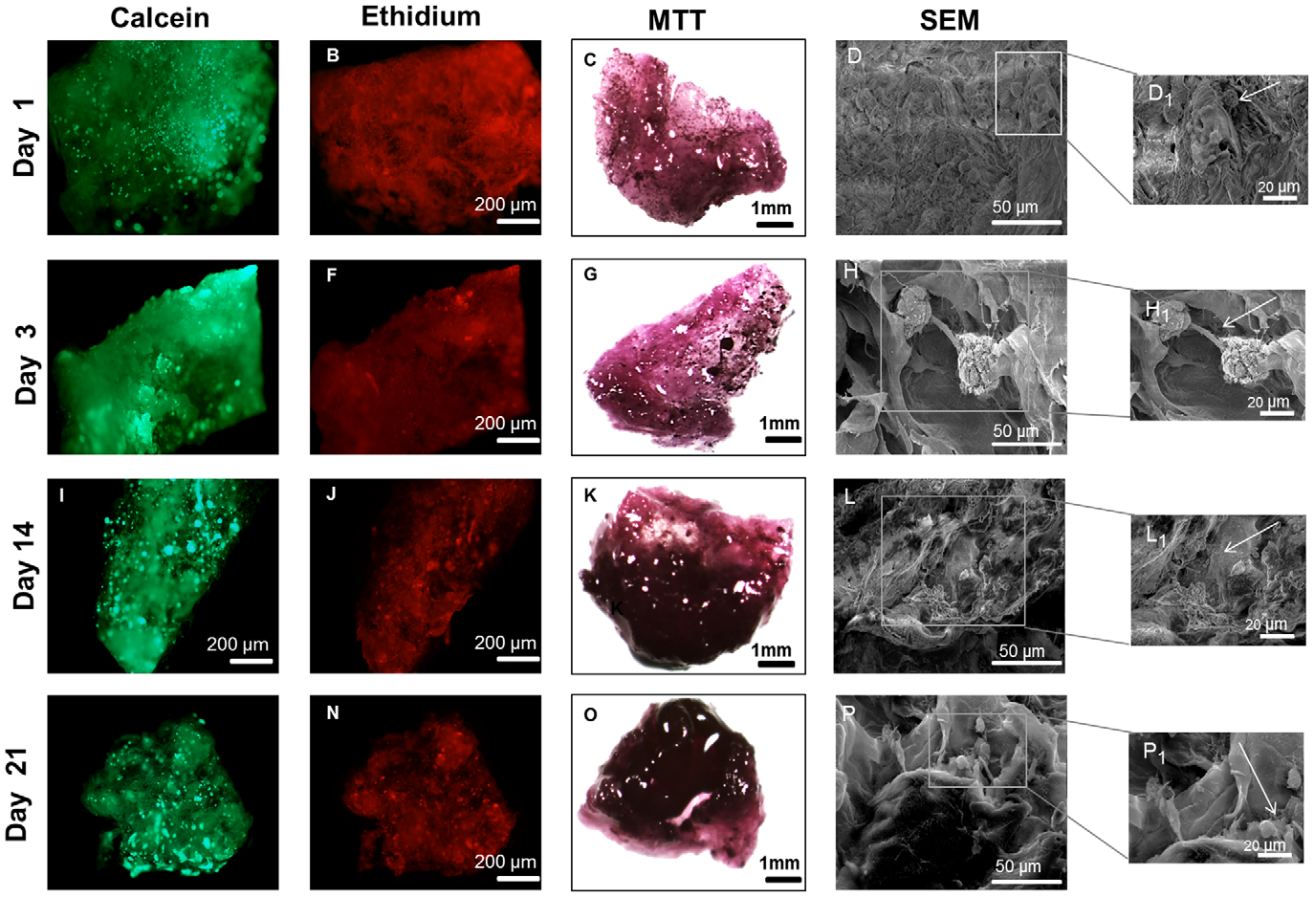
Live/dead assay, MTT assay and cross-section SEM micrographs of BCH seeded on scaffold at day 1(A, B, C), 3(D, E, F), 14 (G, H, I) and 21(J, K, L) of culture in proliferation medium.

**Figure 7:**
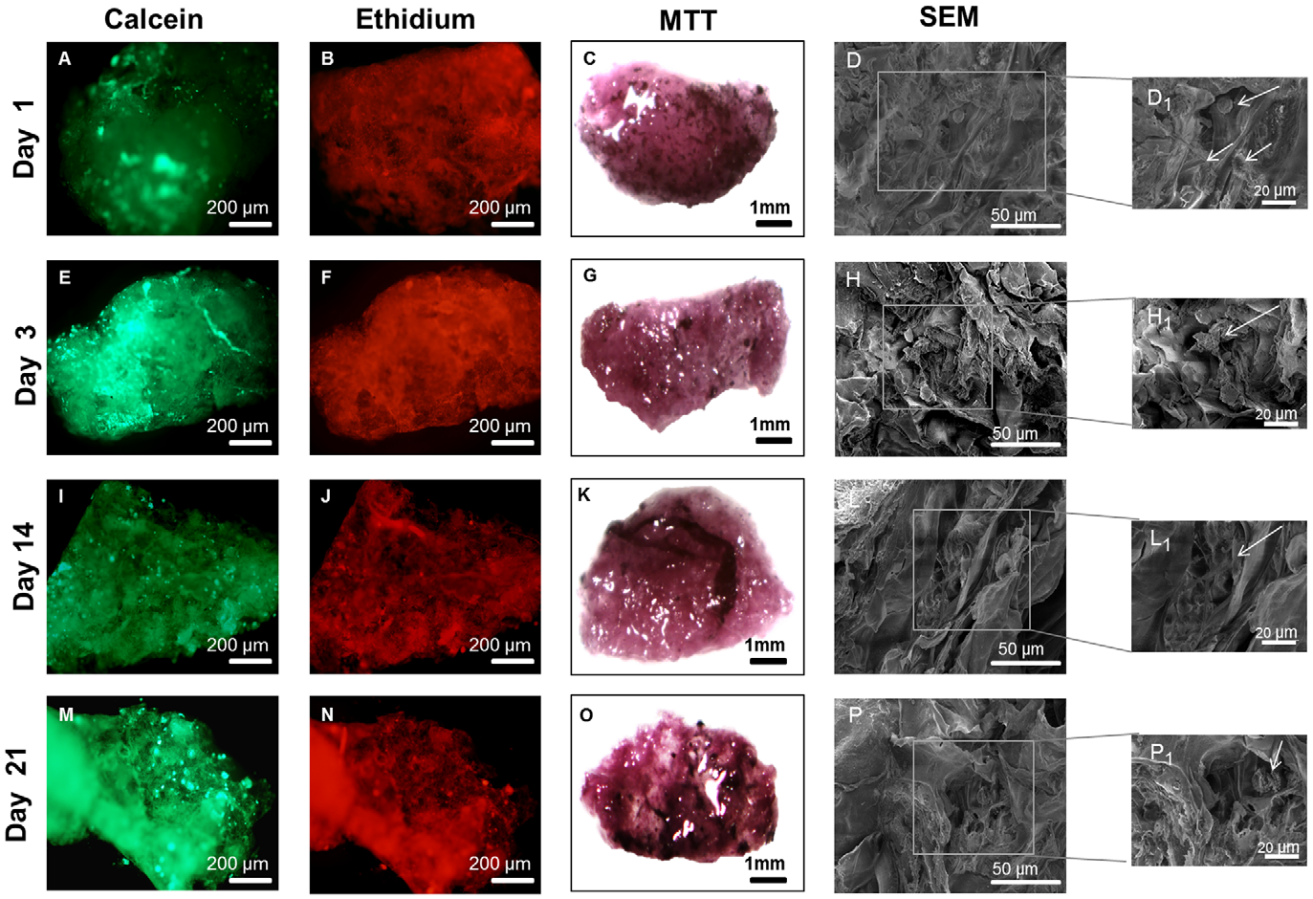
Live/dead assay, MTT assay and cross-section SEM micrographs of hMSCs seeded on scaffold at day 1(A, B, C), 3(D, E, F), 14 (G, H, I) and 21(J, K, L) of culture in proliferation medium.

The cell viability tests with BCH (Figure 6A, 6D, 6G and 6J) and hMSCs (Figure 7A, 7D, 7G and 7J) showed evidence of cell attachment and a large amount of living cells (green)). This was consistent with the results obtained on flat surfaces.. After 1 day it was possible to see that the cells tended to aggregate. Furthermore, the results obtained with MTT assay for BCH (Figure 6B, 6E, 6H and 6K) and hMSCs (Figure 7B, 7E, 7H and 7K) suggested an increase in cell number and metabolic activity due to the increase in dark purple staining over time when compared with the control, the empty scaffold (Figure S1).

Cell adhesion and morphology was further studied by SEM using cross-sections of the scaffolds (Figure 6C, 6F, 6I and 6L). The results obtained for BCH at day 1 showed that cells attached to the surface, displaying a round shape. During the following culture time the adherent cells were more spread out along the scaffolds. The BCH presented a round shape in all time points which was an indication of phenotype retention and essential for matrix deposition [54]. The results for hMSCs (Figure 7C, 7F, 7I and 7L) revealed that the cells were attached to the surface and presented a more stretched morphology. After 1 day, cells started to adhere and an increase in terms of cell migration occurred in the inner areas of the scaffolds (t = 14 days).

#### Cell proliferation

**Figure 8:**
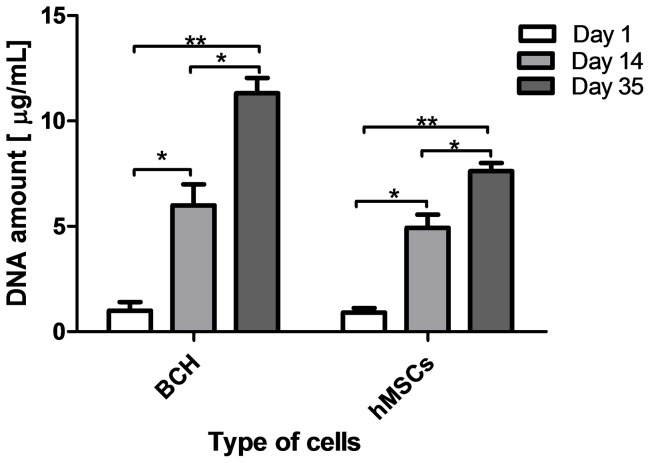
DNA assay on the scaffolds seeded with BCH and hMSCs in differentiation medium. Significant differences between each cell type at different time points were found for p<0.05(*) and p<0.01(**).

Cell proliferation in differentiation medium was evaluated using a DNA assay (Figure 8). The result obtained for the two types of cell showed that the number of both types of cells increased with increasing culture time. Between the first day of culture and after 35 days there were significant differences in the amount of BCH and hMSCs, indicating that the cells continued proliferation even after long time culture which corroborated the results of cell viability tests.

#### Histology

**Figure 9:**
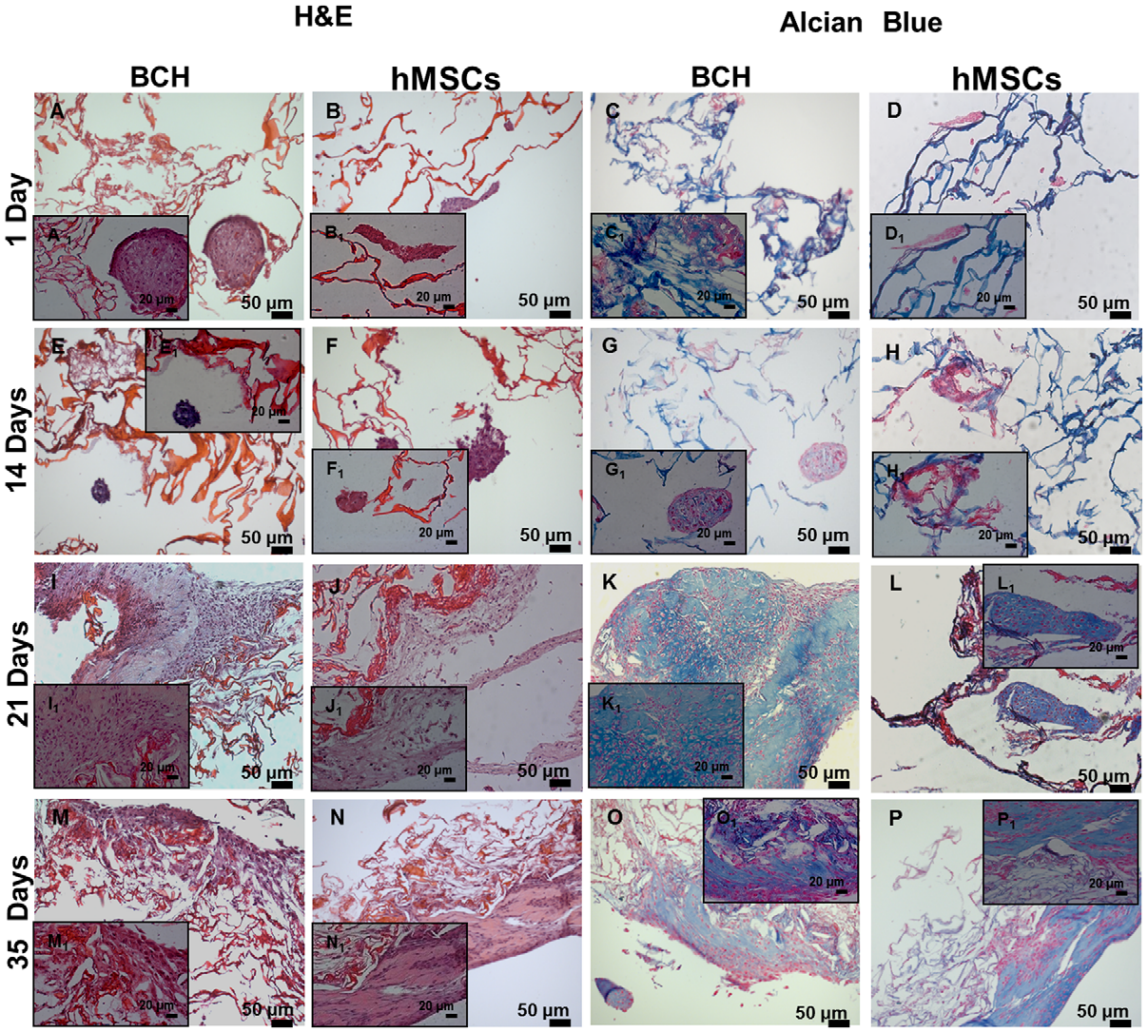
Histological cross-sections of scaffolds seeded with BCH and hMSCs stained by H&E and Alcian blue at different days of culture in differentiation medium.

Cell distribution and matrix production in differentiation medium was evaluated using histology cross-sections stained with H&E and alcian blue (Figure 9).

The H&E staining of scaffold seeded with BCH showed the round morphology of cells. Moreover, over time the abundance of cells per section was increased in support of cell proliferation. At day 1, cells started to attach to the walls forming small aggregates. At day 14 the size of BCH agglomerates increased. During the following weeks, the cells presented a higher dispersion and distribution in the scaffolds. Sulphated GAGs, indicating new cartilage matrix formation, were stained by alcian blue. CS, which gave as well a positive staining for GAGs, can be distinguished from newly deposited matrix by comparing alcian blue staining at day 1 with later time points. Secretion of GAGs by BCH was first observed from 14 days of culture. GAG production increased during subsequent weeks. Lacunae formation was also seen in the matrix surrounding BCH, namely at day 21 and 35. The maintenance of chondrogenic phenotype is indicated by the lacunae formation.

In scaffolds seeded with hMSCs it was possible to see some agglomerated cells at day 1 and after 2 weeks the size of these agglomerates increased (Figure 9). During the following time points the hMSCs were more spread throughout the scaffold. GAG deposition was also assessed and at day 14 a small amount of deposition could be seen. The amount of deposition increased during the next weeks. The deposition of GAGs by hMSCs indicated the chondrogenic differentiation of these cells. The driving force for differentiation in this assay was TGF-β, although a role of CS in chondrogenic differentiation cannot be excluded [35].

## Conclusions

Flat CHT/CS PEMs prepared using LbL elicit a positive effect on BCH cells, allowing its attachment and proliferation. It was possible to use LbL combined with spherical template leaching to produce an innovative 3D nanostructured constructs of CHT/CS with high porosity and interconnectivity, just composed by self-assembled multilayers of these polyelectrolytes. Both BCH and hMSCs could adhere and proliferate in these scaffolds, Secretion of GAGs was observed in BCH and hMSCs upon culture in chondrogenic differentiation medium, indicating that the chondrogenic phenotype was maintained and hMSCs differentiation was successfully induced. Our results suggest that nanostructured scaffolds of chitosan and chondroitin sulphate obtained by LbL technology could have potential use in TE approaches for cartilage namely for matrix blunt and partial thickness/chondral defects. The scaffold would be implant after cell culture *in vitro* in order to increase the production of matrix that will start under static conditions after 14 days. The use of low oxygen tension, mechanical stimulation could accelerate this process [55]. As future work we envisage the production of scaffolds with an increase in terms of mechanical properties by incorporation of fillers, such as nanotubes or using crosslinked PEMs that will also reduce its degradation rate. The ability of these PEMs to sustain delivery of growth factors such as TGF-β could be another way to improve its performance. These improvements will open new horizons for clinical application in the field of cartilage tissue engineering.

## Supporting Information

Figure S1 **Empty scaffold stained by MTT assay.**
(TIF)
