# Supplementary figures and images for: Nanostructured 3D Constructs Based on Chitosan and Chondroitin Sulphate Multilayers for Cartilage Tissue Engineering
Source: PLoS One. 2013 Feb 20;8(2):e55451. doi: 10.1371/journal.pone.0055451 (PMC3577876; doi:10.1371/journal.pone.0055451)

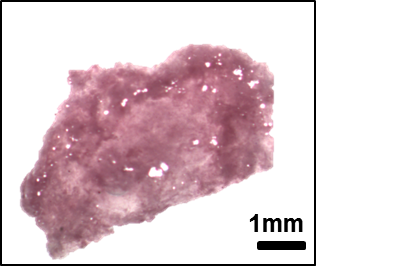

Supplement: Figure S1 — Empty scaffold stained by MTT assay. (TIF) [file pone.0055451.s001.tif]
